# [Expression of Concern] miR-135b, upregulated in breast cancer, promotes cell growth and disrupts the cell cycle by regulating LATS2

**DOI:** 10.3892/ijo.2025.5802

**Published:** 2025-09-15

**Authors:** Kaiyao Hua, Jiali Jin, Junyong Zhao, Jialu Song, Hongming Song, Dengfeng Li, Niraj Maskey, Bingkun Zhao, Chenyang Wu, Hui Xu, Lin Fang

Int J Oncol 48: 1997-2006, 2016; DOI: 10.3892/ijo.2016.3405

Following the publication of the above paper, it was drawn to the Editor's attention by a concerned reader that, for the scratch-wound assay data shown in Fig. 4A, the '24 h/MCF-7/mir-135b inhibitor' data panel looked strikingly similar to the '24 h/MCF-7/NC' data panel in Fig. 10C, albeit the panels were featured rotated through 180° with respect to each other.

The authors were contacted by the Editorial Office to offer an explanation for this possible anomaly in the presentation of the data in this paper, although up to this time, no response from them has been forthcoming. Owing to the fact that the Editorial Office has been made aware of potential issues surrounding the scientific integrity of this paper, we are issuing an Expression of Concern to notify readers of this potential problem while the Editorial Office continues to investigate this matter further.

